# MiR‐17~92 ablation impairs liver regeneration in an estrogen‐dependent manner

**DOI:** 10.1111/jcmm.12782

**Published:** 2016-01-19

**Authors:** Yongjie Zhou, Lei Zhang, Hongjie Ji, Xufeng Lu, Jie Xia, Li Li, Fei Chen, Hong Bu, Yujun Shi

**Affiliations:** ^1^ Laboratory of PathologyWest China HospitalSichuan UniversityChengduChina; ^2^ Key Laboratory of Transplant Engineering and ImmunologyMinistry of HealthChengduChina; ^3^ Department of PathologyWest China HospitalSichuan UniversityChengduChina

**Keywords:** miR‐17˜92, liver regeneration, gender disparity, oestrogen

## Abstract

As one of the most important post‐transcriptional regulators, microRNAs (miRNAs) participate in diverse biological processes, including the regulation of cell proliferation. MiR‐17~92 has been found to act as an oncogene, and it is closely associated with cell proliferation. However, its role in liver regeneration is still unclear. We generated a hepatocyte‐specific miR‐17~92‐deficient mouse and used a mouse model with 70% partial hepatectomy (PH) or intraperitoneal injection of carbon tetrachloride to demonstrate the role of MiR‐17~92 in liver regeneration. In quiescent livers, the expression of the miR‐17~92 cluster showed a gender disparity, with much higher expression in female mice. The expression of four members of this cluster was found to be markedly reduced after 70% PH. The ablation of miR‐17~92 led to obvious regeneration impairment during the early‐stage regeneration in the female mice. Ovariectomy greatly reduced miR‐17~92 expression but significantly promoted liver regeneration in wild‐type mice. In addition, early regeneration impairment in miR‐17~92‐deficient livers could be largely restored following ovariectomy. The proliferation suppressors p21 and Pten were found to be the target effectors of miR‐17~92. MiR‐17~92 disruption resulted in elevated protein levels of p21 and Pten in regenerating livers. MiR‐17~92 functions as a proliferation stimulator and acts in an oestrogen‐dependent manner. The loss of this miRNA results in increases in p21 and Pten expression and therefore impairs liver regeneration in female mice.

## Introduction

The adult liver is unique because of its mysterious intrinsic capacity to regenerate by the proliferation of fully differentiated hepatocytes, which are quiescent and normally divide only once or twice each year in mice and even less frequently in humans 1. However, these cells are able to divide numerous times in response to liver tissue injury or loss. Liver regeneration is a sophisticated process involving a complex network of cytokine and growth factor signalling between hepatocytes and other mesenchymal cells 2. To date, 70% partial hepatectomy (PH) in mice or rats is the most commonly used model for studying liver regeneration. Remnant hepatocytes enter and progress through the cell cycle in a highly synchronized fashion, and the entire process is completed within 7 days 3. Carbon tetrachloride (CCl_4_) and other toxic agents have also been used to assess this process in some studies 4, 5.

MicroRNAs (miRNAs) are a class of small, non‐coding RNAs that silence target mRNAs by binding to their 3′‐untranslated regions (UTRs) 6. They have emerged as important post‐transcriptional regulators in liver physiology and regeneration and in various diseases 7, 8. A large number of studies have demonstrated that many liver pathophysiological processes are highly associated with miRNA dysregulation 9, 10. Of the deregulated miRNAs, the miR‐17~92 cluster has attracted attention because of its paradoxical functions in different tissues and cells 11. MiR‐17~92 functions as a tumour suppressor by decreasing the E2F1 and cyclin D1 protein concentrations in tumour cell lines 12, 13. In contrast, this cluster is up‐regulated to enhance cell growth, promote angiogenesis and inhibit apoptosis in diverse malignancies, including lung cancer and lymphoma 14, 15. In addition, loss‐of‐function of the MiR‐17~92 cluster results in a decrease in embryo size and immediate postnatal death in all animals 16.

The miR‐17~92 cluster encodes seven members, including miR‐17‐5p, miR‐17‐3p, miR‐18a‐5p, miR‐19a‐3p, miR‐19b‐3p, miR‐20a‐5p and miR‐92‐1 17. Recently, several independent studies have shown diverse roles for this cluster in the liver. The overexpression of miR‐17‐5p, a novel prognostic marker for hepatocellular carcinoma (HCC), promotes the migration of HepG2 liver cancer cells. The knockdown of this miRNA leads to a defect in the G1 to S transition 18, 19, 20. MiR‐18a can accelerate cell proliferation by reducing the oestrogen receptor alpha (ERα) protein level, and its high expression is often observed in female liver cancer patients 21. Moreover, miR‐19b inhibits hepatic stellate cell activation and decreases fibrogenesis 22. In accordance with the findings for HCC, the miR‐17~92 level has been found to be altered in the regenerating liver, suggesting its potential role in the regulation of liver regeneration 23. In the present study, we examined the expression of the members of the miR‐17~92 cluster in quiescent and regenerating livers. We generated a hepatic‐specific miR‐17~92 knockout mouse to investigate its role in liver regeneration. Our findings show that this cluster displays a gender disparity in the quiescent liver and that it is markedly reduced in regenerating the livers of female mice. Loss of miR‐17~92 increases the protein levels of p21 and Pten in an oestrogen‐dependent manner. As a result of this loss, liver regeneration is impaired, specifically in female mice.

## Materials and methods

### Animal studies

Hepatocyte‐specific *MiR‐17˜92* cluster knockout mice were generated by crossing *miR‐17˜92* ^flox/flox^ mice with mice expressing Cre recombinase under the control of a rat albumin promoter, as previously described 24. The mice were maintained under alternating 12‐h light/dark cycles, fed regular chow and provided water *ad libitum*. Animal procedures and care were conducted in accordance with the institutional guidelines and in compliance with national and international laws and policies. To assess the role of miR‐17~92 in liver regeneration, we performed 70% PH on 8–12‐week‐old miR‐17~92 conditional knockout (CKO) mice and their sex‐matched wild‐type (WT) littermates. Acute toxic hepatic injury was induced by intraperitoneal injection of 10 ml/kg body weight of a 10% solution of CCl_4_ in olive oil. A single dose of 5‐bromo‐2′‐deoxyuridine (BrdU; Sigma‐Aldrich, St. Louis, MO, USA) was injected intraperitoneally at 50 mg/kg animal weight (10 mg/ml in PBS) at 1 hr prior to sacrifice. Liver specimens were harvested at the indicated time‐points for histological analysis or nucleic acid and protein isolation.

### Reverse transcription and quantitative real‐time PCR

Total RNA was isolated from liver tissues using TRIzol reagent (Invitrogen, San Diego, CA, USA), and cDNA was synthesized by following the manufacturer's protocol (Bio‐Rad, Hercules, CA, USA). Quantitative real‐time PCR (qRT‐PCR) was performed with a standard SYBR green PCR kit (Bio‐Rad), and PCR‐specific amplification was applied in the Bio‐Rad CFX96 real‐time PCR machine. For miRNA quantification, Bulge‐loop miRNA qRT‐PCR Primer Sets (one RT primer and a pair of qPCR primers for each set) specific for the miR‐17~92 cluster were designed by RiboBio (Guangzhou, China). The relative expression of genes, including U6, miR‐17‐3p, miR‐17‐5p, miR‐18a‐5p, miR‐19a‐3p, miR‐19b‐3p, miR‐20a‐5p and miR‐92‐1, was calculated with the 2^−ΔΔCt^ method.

### Histology and immunohistochemistry

Haematoxylin and eosin staining and immunohistochemistry staining for Ki‐67 and BrdU were carried out on 5‐μm‐thick paraffin sections of liver tissues. The percentage of positive nuclei was counted in five consecutive high‐power fields.

### Cell culture

The human liver cancer cell line HepG2 was maintained in DMEM supplemented with 10% fetal bovine serum, 2 mM glutamine, 100 U/ml penicillin and 100 μg/ml streptomycin. The cells were maintained at 37°C in a 5% (v/v) CO_2_ atmosphere. When 60–70% confluent, oestrogen (E_2_) was added to the culture medium to a final concentration of 10 nM, and after 24 hrs stimulation, cells were harvested for further investigation 25.

### Luciferase assay

The 3′UTRs of mouse Pten and p21 were amplified from genomic DNA using two primer sets (forward primer, 5′‐GTGGAGACAGGCTGAT‐3′; reverse primer, 5′‐ATGCCTTATTATTCGTG‐3′ for Pten; and forward primer, 5′‐CCCCAGCCCAAACAAAGA‐3′; reverse primer, 5′‐GGGTTTCGGTTGGTCCTCT‐3′ for p21), which contain binding sites for the miR‐17~92 cluster and are conserved in humans. Mutagenesis of these binding sites was carried out using a Quick Change XL mutagenesis kit (Agilent Technologies, Santa Clara, CA, USA). The WT Pten and p21 3′UTRs, as well as the mutated 3′UTR fragments, were each cloned downstream from a firefly luciferase reporter. These firefly luciferase constructs contained either a WT or mutated 3′UTR. Each construct was transfected into a Hep1‐6 cell line, together with a Renilla luciferase construct for normalization control, 20/40 nM miRNA mimics (mix of mir‐17‐5p, mir‐19a‐3p, miR‐19b‐5p and miR‐20a‐5p) or a control mimic, mir‐1 respectively. The luciferase activity of each construct was determined by dual luciferase assay (Promega, Madison, WI, USA) at 48 hrs post‐transfection.

### Western blotting

Liver samples were homogenized in Radio Immunoprecipitation Assay (RIPA) lysis buffer with phenylmethanesulfonyl fluoride (Beyotime, Shanghai, China), incubated on ice for 1 hr and centrifuged for 15 min. at 12,000 × g at 4°C. Supernatants were collected. The protein concentration was measured by bovine serum albumin assay (Cowin Biotech, Beijing, China). For western blotting, 100 μg total protein per lane was used to examine the expression level of the target protein. Mouse anti‐p21 (Santa Cruz, Dallas, TX, USA), rabbit anti‐Pten, cyclin D1, CDK1, CDK2, CDK4, cyclin A, cyclin B and AKT (Abcam, Cambridge, CA, USA) antibodies were used. ECL reagent was used for chemiluminescence detection. Signal intensities were quantified and normalized to the histone H3 or GAPDH intensity using ImageJ software.1

### Statistical analysis

All data were expressed as the mean ± S.D. SPSS statistical package was used (version 13.0; SPSS Inc., Chicago, IL, USA) for statistical analysis. Significance was determined with two‐tailed Student's *t*‐test. A *P* < 0.05 was considered significant.

## Results

### MiR‐17~92 expression in quiescent and regenerating livers

**Figure 1:**
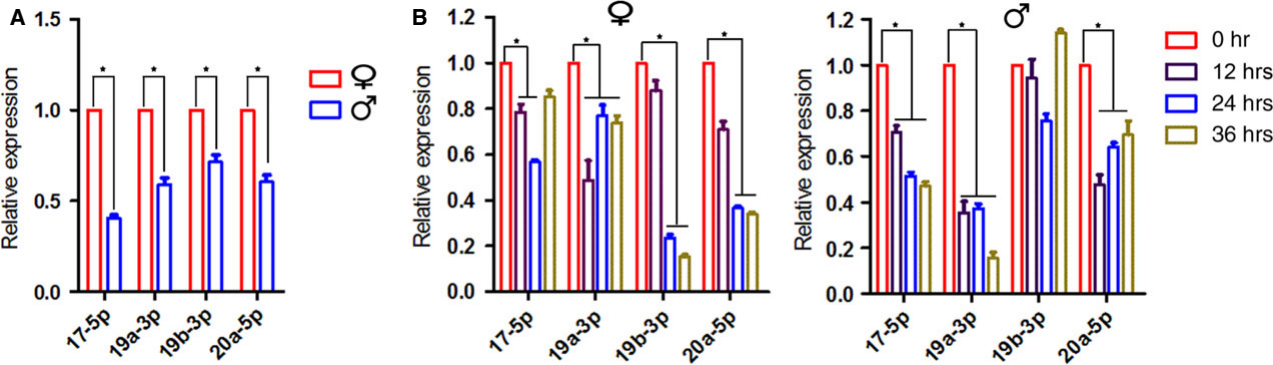
MiR‐17~92 expression in quiescent and regenerating livers. (**A**) The expression levels of four members of the miR‐17~92 cluster show noticeable gender disparity in the quiescent liver. (**B**) The expression of each of the members is significantly reduced in the regenerating livers of mice of both genders. The data represent the mean ± S.D.; *n* = 5; *P < 0.05.

First, we examined miR‐17~92 expression in the quiescent livers. Of the seven family members, miR‐92‐1, miR‐18a‐5p and miR‐17‐3p expression was quite low. We then focused on the other four members, including miR‐17~5p, miR‐19a‐3p, miR‐19b‐3p and miR‐20a, the expression of which differed between the genders, with higher expression in the female mice (Fig. 1A). Notably, expression of miR‐17‐5p, the most abundant member in liver tissue, was approximately 2.5‐fold higher in the female mice than in their male counterparts.

To determine whether miR‐17~92 participates in liver regeneration, we examined the expression levels of these four members during the early proliferating stage after PH. These miRNAs were found to exhibit decreases in the expression of varying degrees in the mice of both genders following 70% PH (Fig. 1B and C).

### Generation of miR‐17~92‐deficient mice

**Figure 2:**
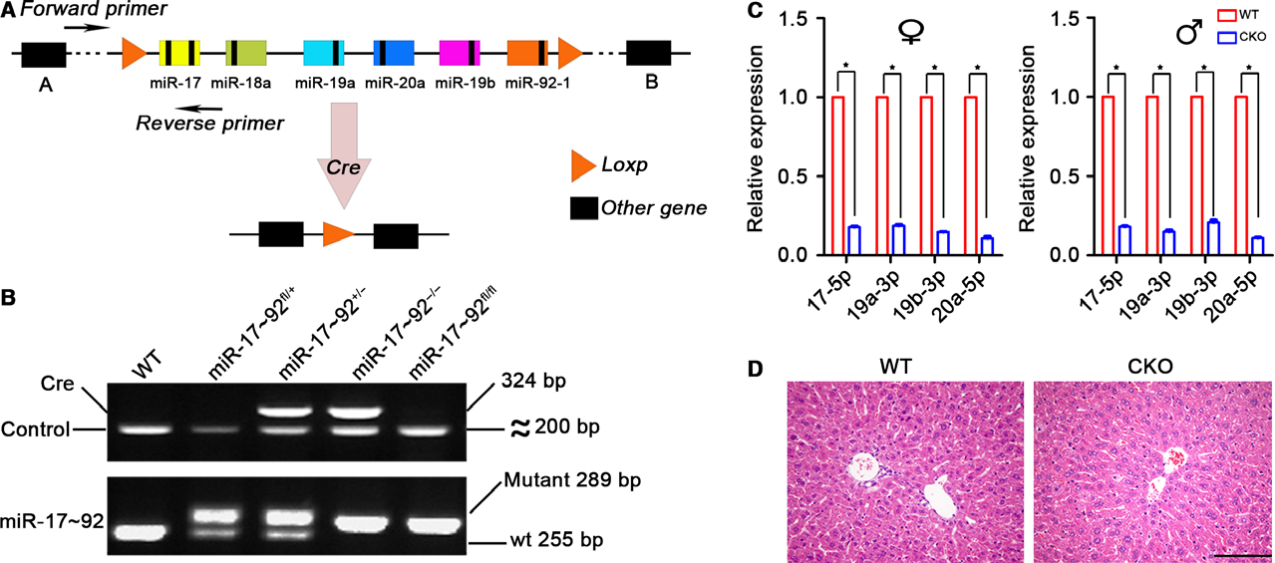
Confirmation of the hepatocyte‐specific deletion of miR‐17~92. (**A**) A brief schematic diagram of the construction of miR‐17~92 conditional knockout mice. (**B**) An electropherogram of PCR‐amplified tail DNA. The Apo gene was used as a positive control for Cre recombinase. (**C**) Knockout efficiency was determined by qRT‐PCR. The expression level of each member of the miR‐17~92 cluster was set at 1 in the control mice. The data represent the mean ± S.D.; *n* = 5. (**D**) Haematoxylin‐eosin staining of the liver tissues reveals no noticeable hepatic histological abnormalities in adult miR‐17~92^−/−^ mice, scale bar: 100 μm.

We generated hepatocyte‐specific miR‐17~92 knockout mice, and their genotypes were assessed by PCR analysis of tail DNA (Fig. 2A and B). Each individual member of the miR‐17~92 cluster displayed a 70–90% reduction in expression in the mutant livers of the adult mice. No expression changes were found in the other organs evaluated (Fig. 2C, Fig. S1).

MiR‐17~92 CKO mice were born at the expected Mendelian ratio, and they were fertile and appeared healthy. All serum parameters were within the normal ranges (Table S1). The mutant liver did not exhibit any obvious histological abnormalities (Fig. 2D).

### Loss of miR‐17~92 impairs early liver regeneration in female mice

**Figure 3:**
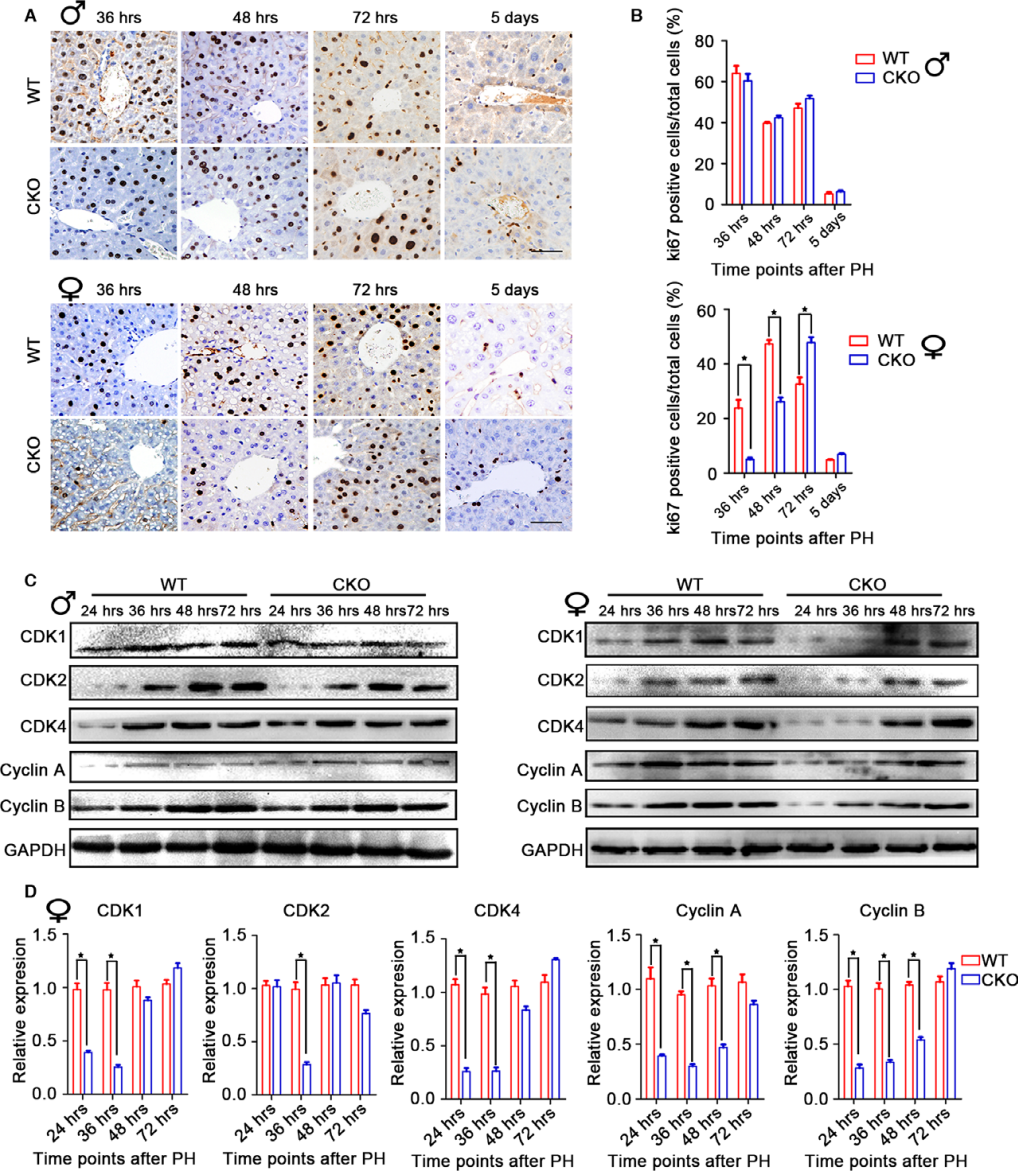
MiR‐17~92 deletion causes an obvious delay in early liver regeneration in female mice after 70% partial hepatectomy. (**A** and **B**) Immunohistochemistry and quantification of Ki67 show that in male mice, miR‐17~92 ablation does not lead to any evident differences in liver regeneration. However, female miR‐17~92^−/−^ mice exhibit remarkably delayed regeneration at the early time‐points. The data represent the mean ± S.D.; *n* = 5; *P < 0.05; scale bar: 50 μm. (**C**) Immunoblotting of cyclins and cell cycle‐dependent kinases (CDKs) at the indicated time‐points in female regenerating liver tissue. GAPDH was used as a loading control. (**D**) The levels of the included proteins are expressed as ratios to GAPDH. The data represent the mean ± S.D.; *n* = 3; *P < 0.05.

To determine the role of miR‐17~92 in liver regeneration, we performed 70% PH on the miR‐17~92 CKO mice and their gender‐matched littermates. No obvious differences were found between the male mice with the different genotypes at each time‐point, as indicated by Ki67 immunoreactivity and BrdU incorporation (Fig. 3A and B and Figs S2 and S3). In addition, immunoblotting of cell cycle markers, including CDK1, CDK2, CDK4, cyclin A and cyclin E, revealed few differences between the WT and mutant livers of the male mice (Fig. 3C). Our data indicate that in male mice, miR‐17~92 has little effect on DNA synthesis or cell proliferation during liver regeneration.

The gender disparity in miR‐17~92 expression prompted us to speculate whether miR‐17~92 ablation has an effect on liver regeneration in female mice. As expected, the proportion of mitotic hepatocytes in the CKO mice was reduced by approximately 80% at 36 hrs and by 50% at 48 hrs after PH (Fig. 3A and B and Fig. S2). These findings were confirmed by the delay in liver reconstitution, as demonstrated by the lower liver weight/bw ratio (Fig. S2). Furthermore, immunoblotting analysis revealed substantial reductions in the levels of cell cycle proteins in the female CKO mice at 24 and 36 hrs (Fig. 3C and D).

We next assessed the liver repair rate in the female mice in response to the acute toxic injury induced by CCl_4_ injection. The ratio of Ki67‐positive cells in the mutant liver was lower at 24 and 36 hrs, in accordance with the results obtained with the surgery model. However, this ratio was higher at 72 hrs compared with that in the WT liver (Fig. S4).

### MiR‐17~92 promotes oestrogen‐dependent liver regeneration

**Figure 4:**
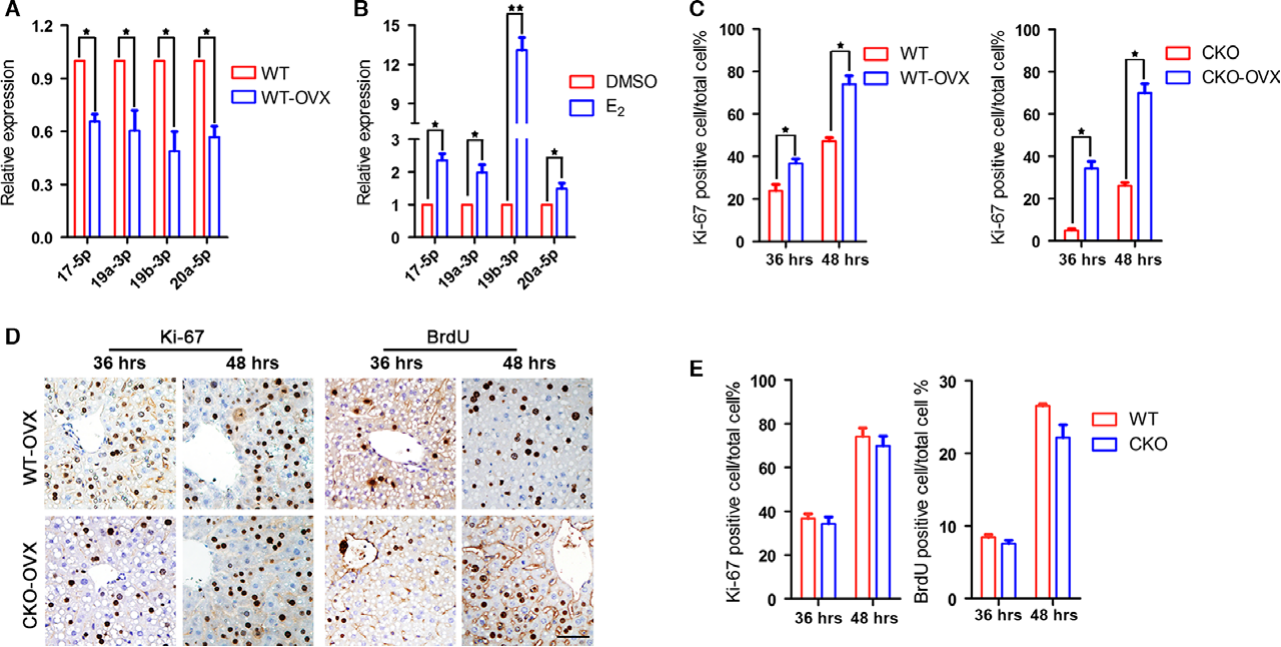
Ovariectomy enhances liver regeneration. (**A**) Ovariectomy reduces the levels of the miR‐17~92 cluster members in WT mice. (**B**) Oestrogen (E2) stimulation promotes miR‐17~92 production in cultured HepG2 cells. (**C**) Liver regeneration is promoted in female mice with both genotypes in response to ovariectomy, as determined by the number of Ki67‐positive hepatocytes. (**D** and **E**) Both Ki67 immunoreactivity and BrdU incorporation indicate that there is no difference in the regeneration rate between the WT and CKO mice after ovariectomy. *P < 0.05, scale bar: 50 μm.

The observation that miR‐17~92 deficiency specifically delayed early liver regeneration in the female mice prompted us to investigate the association between miR‐17~92 and oestrogen. Upon 17‐β‐estradiol (E2) binding, ERs prime gene transcription by interacting directly or indirectly with specific oestrogen response elements (EREs) located in the promoter or enhancer regions of their target genes 25, 26. To elucidate whether the effect of miR‐17~92 on liver regeneration is regulated by oestrogen, we performed ovariectomy (OVX) on female mice and examined the levels of the miR‐17~92 members compared with those in WT mice. To our surprise, the levels of all four members were decreased by 30–40% at 1 month after OVX (Fig. 4A). The production of these members was markedly increased in cultured HepG2 cells after 24 hrs of oestrogen stimulation (Fig. 4B).

We next examined the liver regeneration rate in the OVX mice. After surgery, the mice with both genotypes displayed noticeably elevated liver regeneration rates (Fig. 4C), indicating that oestrogen functions as a proliferation inhibitor. Most intriguingly, the two groups of mice showed similar regeneration rates, as indicated by Ki67 immunoreactivity and BrdU incorporation (Fig. 4D and E). Our data again suggest that miR‐17~92 plays an oestrogen‐dependent role in liver regeneration.

### MiR‐17~92 reduces p21 and Pten

**Figure 5:**
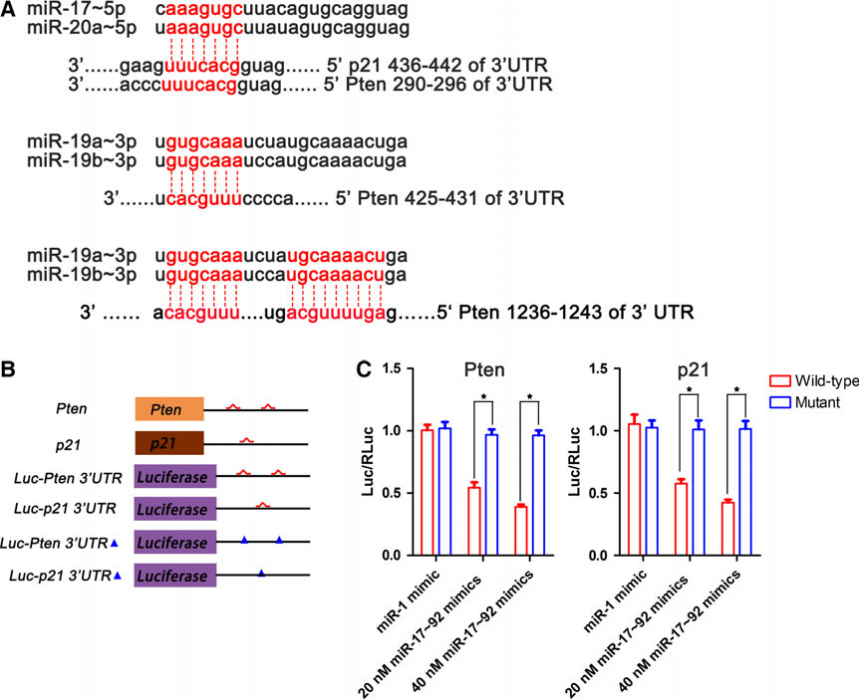
MiR‐17~92 targets Pten and p21. (**A**) Prediction of the binding sites of miR‐17~92 on the 3′UTRs of the Pten and p21 genes using the Targetscan online website. (**B**) Schematic representations of the 3′UTRs of the Pten and p21 genes and their WT miR‐17~92 (red) and mutant binding sites (blue triangle), which were cloned downstream from a luciferase reporter (Luc). (**C**) Specific repression of the Luc‐Pten and p21 3′UTR reporters with miR‐17~92 mimics. The mimic miR‐1 was used as a control, *P < 0.05.

**Figure 6:**
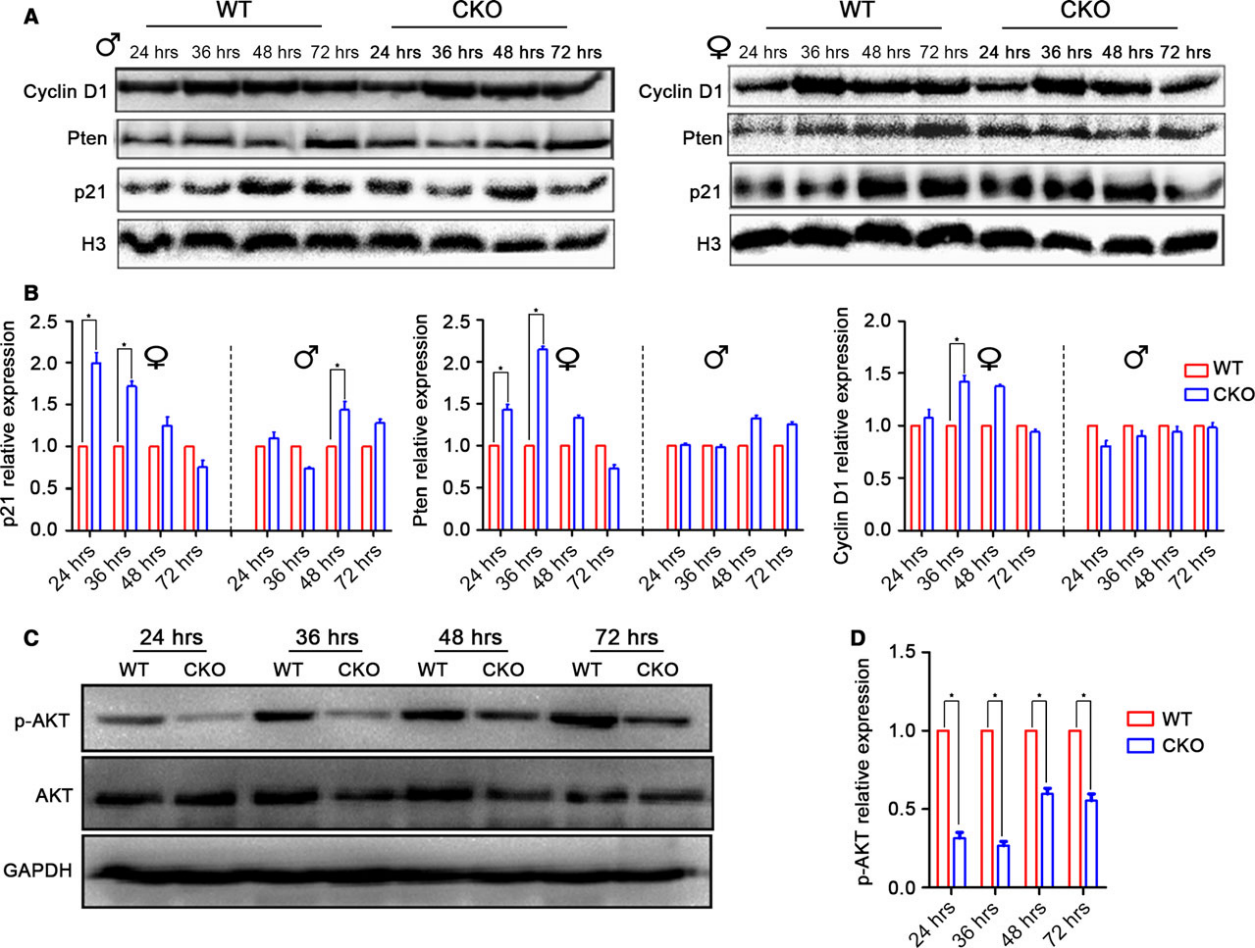
MiR‐17~92 promotes cell proliferation by inhibiting p21 and Pten expression in the regenerating liver. (**A**) Immunoblotting of cyclin D1, Pten, and p21 in regenerating livers of male and female mice. Histone H3 was used as a loading control. (**B**) The relative fold changes of cyclin D1, Pten and p21 in CKO and WT mice. The data represent the mean ± S.D.; *n* = 3; *P < 0.05. (**C** and **D**) Immunoblotting of p‐Akt was substantially reduced in the regenerating livers of the female CKO mice, especially at 24 and 36 hrs after 70% PH. The data represent the mean ± S.D.; *n* = 3; *P < 0.05.

Oestrogen has been reported to enhance miR‐17~92 expression and to act as a tumour repressor in MCF‐7 breast cancer cells 25. In contrast, in our study, miR‐17~92 seemed to promote liver regeneration because its ablation impaired early liver regeneration in the female mice. The potential target effector of miR‐17~92 in this process is still unknown. Using TargetScan and PicTar online software, we found that cyclin D1, the tumour repressor gene Pten, and p21 (also known as the negative cell cycle regulator‐cyclin‐dependent kinase inhibitor CDKN1A) were candidate targets of the miR‐17~92 cluster (Fig. 5A). Luciferase reporter gene assay confirmed that the miR‐17~92 cluster directly targets the 3′UTRs of the Pten and p21 genes, as shown in Figure 5B and C in accordance with previous reports 17, 27. Our immunoblotting results revealed that the loss of miR‐17~92 caused slight changes in the levels of cyclin D1, Pten and p21 levels in the male mice (Fig. 6A and B). In contrast, despite the up‐regulation of cyclin D1, which plays a positive role in accelerating cell proliferation, the levels of both inhibitory Pten and p21 were dramatically increased in the mutant female mice (Fig. 6A and B). Previous studies have demonstrated that p21 inhibits DNA synthesis and results in a delay in the G1–S transition in the regenerating liver 28, 29. Meanwhile, Pten represses the AKT/PI3K signalling cascade, functioning as a regeneration suppressor 30, 31, 32. Indeed, we found that the loss of miR‐17~92 noticeably reduced AKT phosphorylation in the female mice, particularly at 24 and 36 hrs post‐operation (Fig. 6C and D), suggesting that the inhibitory effects of Pten and p21 on liver regeneration overwhelmed the inductive effect of cyclin D1 in association with miR‐17~92 deficiency.

## Discussion

In this study, we observed that the expression and function of miR‐17~92 were oestrogen dependent. In female mice, the miR‐17~92 cluster was much more highly expressed. The loss of this cluster resulted in more significant impairment during the early stage of liver regeneration in the females compared with their male counterparts. Furthermore, we have reported that miR‐17~92 targets Pten and p21, both of which are well known proliferation inhibitors. The disruption of miR‐17~92 increases Pten and p21 expression and reduces AKT phosphorylation, resulting in the impairment of liver regeneration.

The role of oestrogen‐regulated miRNA expression, the target genes of these miRNAs, and the role of miRNAs in health and disease is a ‘hot’ area of research that will yield new insight into the molecular mechanisms behind the relationship between oestrogen action and miRNA. For miR‐17~92, various research groups have found that these miRNAs are regulated by oestrogen stimuli *in vivo* and *in vitro* 25, 33, 34, 35. The molecule basis of the phenomenon has been described above for the co‐regulation effects of activated ESα (oestrogen‐binding ESα) and transcription factor c‐myc 25, 36, 37. Based on our results, we added new evidence that the expression of miR‐17~92 was modulated by oestrogen and then showed sex disparity in the liver.

The regeneration process is precisely controlled by the transcriptional and post‐transcriptional modulation of various genes. As key post‐transcriptional regulators, miRNAs have critical roles in the regeneration process by regulating target genes 8. The decrease in miR‐17~92 in the regenerating livers indicated that this miRNA might act as a negative regulator of proliferation. However, our finding that miR‐17~92 deficiency markedly suppressed early liver regeneration in the female mice but not in the OVX mice or male mice suggests that this miRNA acts as a stimulator of cell proliferation in an oestrogen‐dependent manner. The role of oestrogen in liver regeneration is still contradictory. It is thought to decrease IL‐6, which is a critical cytokine that activates approximately one‐third of the immediate early‐phase genes after PH and is fundamentally important for liver repair and hepatocarcinogenesis 38, 39. The lower serum IL‐6 concentration in the presence of oestrogen might be responsible for the low regeneration rate and low HCC incidence that have been reported in female animals and humans. In contrast, other studies have reported that the serum oestrogen level and number of activated ERs in nuclei are elevated after 70% PH in rats. Therefore, oestrogen is regarded as a stimulating factor that promotes liver regeneration by enhancing DNA synthesis 40, 41, 42. These inconsistent findings do not clarify the true function of oestrogen in liver regeneration. In this study, we found that OVX significantly enhanced proliferation, regardless of whether the mice were WT or mutant. This information provides new evidence that oestrogen slows liver regeneration.

Our *in vivo* and *in vitro* experiments revealed that oestrogen induced miR‐17~92 expression. It would be interesting to determine why oestrogen inhibits liver regeneration while miR‐17~92 promotes this process in an oestrogen‐dependent manner. Two EREs have been identified in the miR‐17~92 gene promoter, offering additional evidence that oestrogen regulates miR‐17~92. Most intriguingly, high miR‐17~92 expression can, in turn, suppress the oestrogen/ERα signalling pathway by targeting ERα and its coupled protein, AIB1 25.

The interdependence of miR‐17~92 and oestrogen in the control of cell proliferation prompted us to investigate the target factors that directly modulate cell cycle progression. We assumed that the inhibitory effect of oestrogen on liver regeneration was at least partly offset by the presence of miR‐17~92. In our study, the ablation of miR‐17~92 increases the expression of regeneration inhibitors and therefore impairs liver regeneration. Numerous studies have shed light on the role of miR‐17~92 in regulating cell proliferation by modulating target gene translation. In this study, we identified cyclin D, p21 and Pten as target genes of miR‐17~92. Both the Pten and p21 proteins were noticeably increased following miR‐17~92 inactivation, indicating that this miRNA might promote liver regeneration and that this activity might be partly dependent on Pten and p21 degradation. MiR‐17~92 has also been found to be tightly linked to the E2F family members, critical transcription factors that drive the G1/S phase transition in mitotic cells 36, 43. Considering that the loss of E2F1 has little effect on liver regeneration or hepatocarcinogenesis in mice 44, we did not examine E2F1 in response to miR‐17~92 ablation.

**Figure 7:**
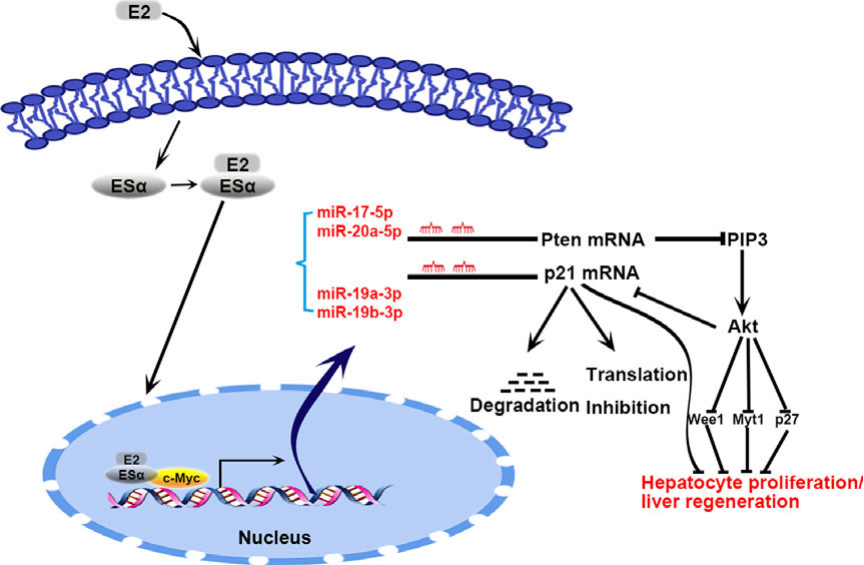
Proposed mechanism of miR‐17~92‐mediated liver regeneration facilitated by the inhibition of p21 and Pten.

In summary, our study has demonstrated for the first time that the miR‐17~92 cluster promotes liver regeneration in an oestrogen‐dependent manner. As summarized in Figure 7, a high oestrogen level slows liver regeneration. At the same time, oestrogen stimulates the production of miR‐17~92, which, in turn, enhances hepatocyte proliferation through Pten and p21 degradation. As a result, the negative effect of oestrogen on regeneration is partly offset by miR‐17~92. Our work provides new insights into the complicated roles of oestrogen and miRNAs in regulating liver regeneration. This information may facilitate the development of novel strategies for intervening in cases of aberrant liver regeneration.

## Conflicts of interest

The authors report no potential conflicts of interest.

## Author contribution

Yongjie Zhou, Lei Zhang, Hongjie Ji, Li Li and Fei Chen performed the research; Yongjie Zhou, Jie Xia, Hongjie Ji and Xufeng Lu analysed the data and Yongjie Zhou, Hong Bu and Yujun Shi designed the study and wrote the paper.

## Supporting information


**Figure S1** The expression of miR‐17~92 cluster in heart, lung, kidney and intestine was examined using qRT‐PCR. The data represent the mean ± S.D.; *n* = 3.
**Figure S2** BrdU incorporation assay showed that there was no significant difference of regeneration rate between the male mice of two genotypes at the indicated time‐points (**A** and **B**).
**Figure S3** Proliferating liver sample slides were co‐stained with cell mitosis marker ki‐67 (green), hepatocyte‐specific marker albumin (red) and DAPI (blue).
**Figure S4** Disruption of miR‐17~92 led to an obvious regeneration impair in CCl4 treated female mice.
**Table S1** Liver‐Specific Serum Markers in WT and miR‐17~92−/− Mice.
