# Supplementary material for: MiR‐17~92 ablation impairs liver regeneration in an estrogen‐dependent manner
Source: J Cell Mol Med. 2016 Jan 19;20(5):939–48. doi: 10.1111/jcmm.12782 (PMC4831359; doi:10.1111/jcmm.12782)
Supplement: Supplementary file 1 — Figure S1 The expression of miR‐17~92 cluster in heart, lung, kidney and intestine was examined using qRT‐PCR. The data represent the mean ± S.D.; n = 3. Figure S2 BrdU incorporation assay showed that there was no significant difference of regeneration rate between the male mice of two genotypes at the indicated time‐points (A and B). Figure S3 Proliferating liver sample slides were co‐stained with cell mitosis marker ki‐67 (green), hepatocyte‐specific marker albumin (red) and DAPI (blue). Figure S4 Disruption of miR‐17~92 led to an obvious regeneration impair in CCl4 treated female mice. Table S1 Liver‐Specific Serum Markers in WT and miR‐17~92−/− Mice. [file JCMM-20-939-s001.docx]

**Supplementary table S1: Liver-Specific Serum Markers in WT and miR-17~92^-/-^ Mice**

| Serum Marker | WT Mice (n=8) | MiR-17~92^-/-^ Mice (n=8) |
| --- | --- | --- |
| Aspartate transaminase level, IU/L | 31.75(9.75) | 28(6.36) |
| Alanine transaminase level, IU/L | 29(9.16) | 29.75(11) |
| Total cholesterol measurement, mmol/L | 88.5(9.89) | 83.75(9.79) |
| Triglyceride level, mg/dL | 76.5(7.38) | 78.75(6.73) |
| Total bilirubin level, mg/dL | 0.275(0.07) | 0.25(0.09) |


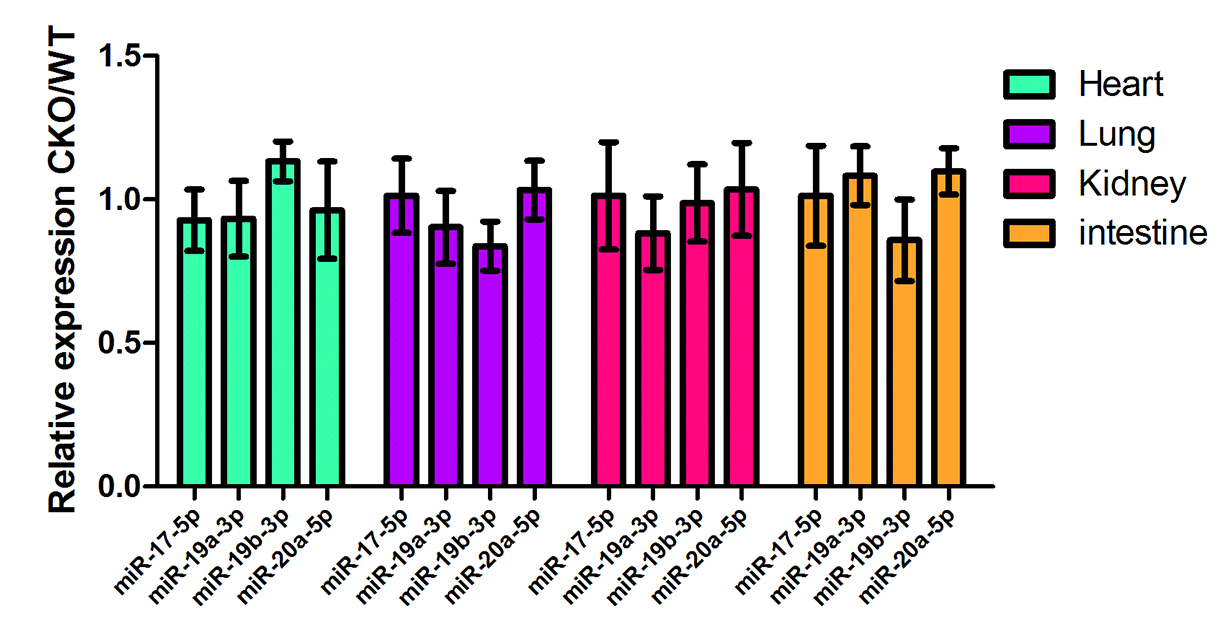


**Supplementary figure S1.** The expression of miR-17~92 cluster in heart, lung, kidney, and intestine was examined using qRT-PCR. The data represent the mean ± SD; n = 3.


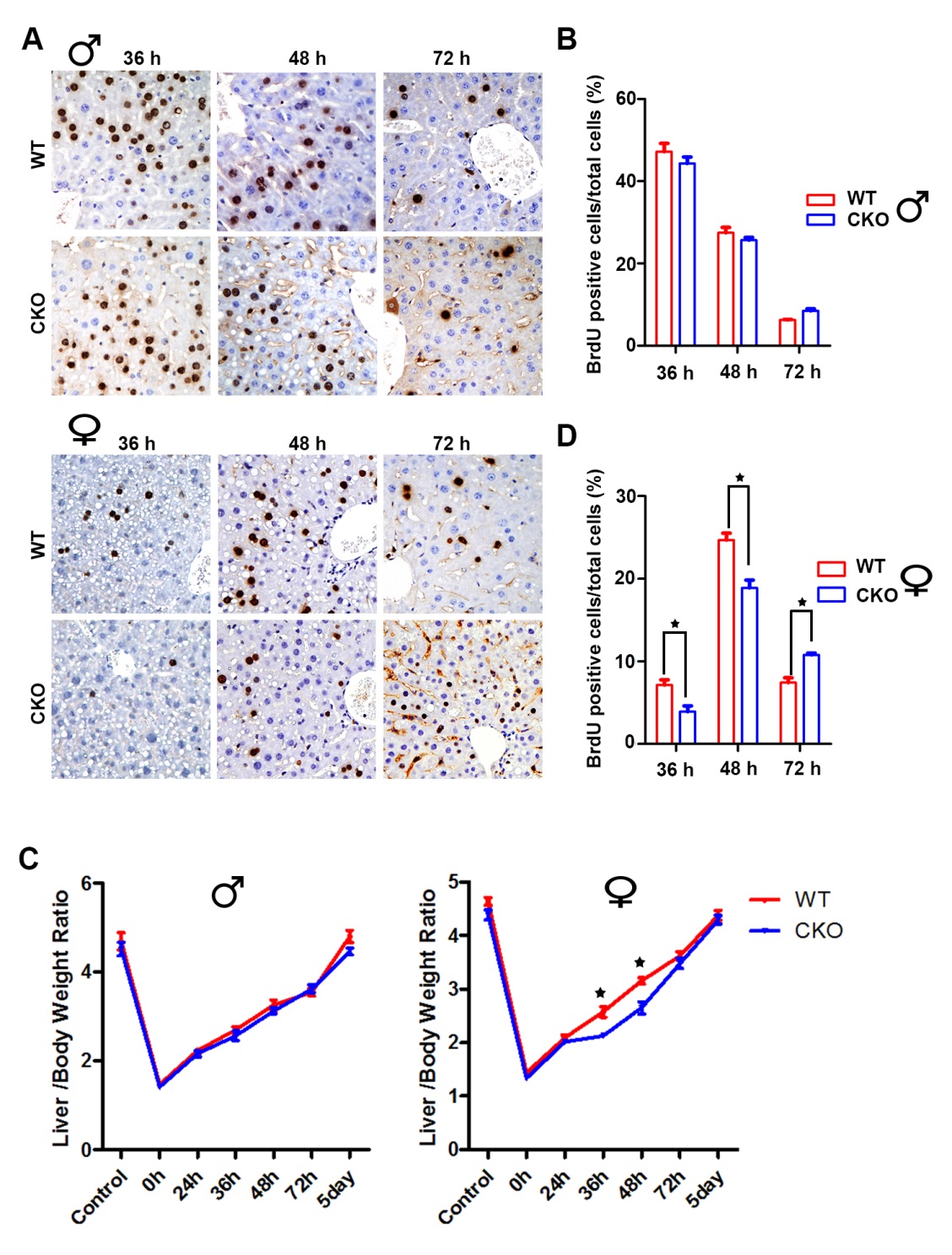


**Supplementary figure S2.** BrdU incorporation assay showed that there was no significant difference of regeneration rate between the male mice of two genotypes at indicated time points (A and B). On the contrary, obviously impaired early regeneration was observed in female mutant mice (A and B). The ratios of liver weight/body weight (LW/BW) were calculated at different time points after PH in male and female mice (C). The data represent the mean ± SD; n = 5; *P<0.05.


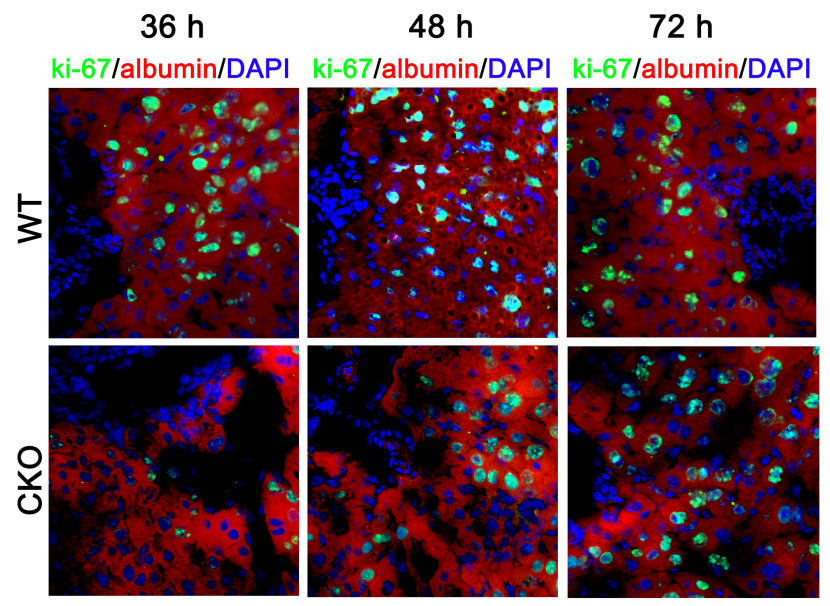


**Supplementary figure S3.** Proliferating liver sample slides were co-stained with cell mitosis marker ki-67 (green), hepatocyte specific marker albumin (red) and DAPI (blue). The results indicated that the proliferating cells at these time points are mainly albumin-expressing hepatocytes.


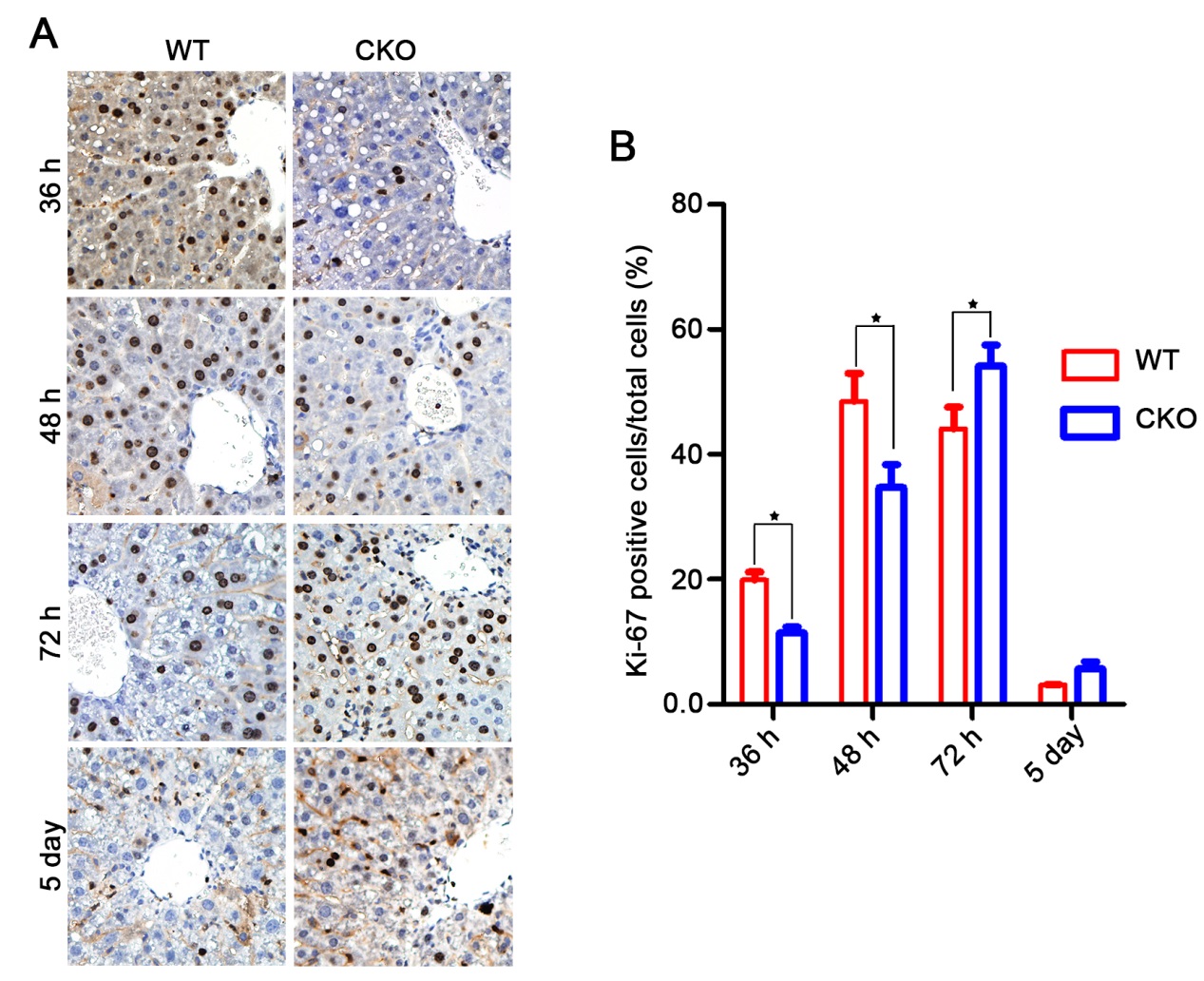


**Supplementary figure S4.** Disruption of miR-17~92 led to an obvious regeneration impair in CCl4 treated female mice. (A) Ki67 immunohistochemistry at indicated time points. (B) Calculation of the rate of Ki67-positive hepatocytes at different time points after PH. The data represent the mean ± SD; n = 5. **P* < 0.05.
